# Biotechnology systems engineering: preparing the next generation of bioengineers

**DOI:** 10.3389/fsysb.2025.1583534

**Published:** 2025-04-29

**Authors:** Sebastián Espinel-Ríos

**Affiliations:** Department of Chemical and Biological Engineering, Princeton University, Princeton, NJ, United States

**Keywords:** biotechnology systems engineering, process systems engineering, systems biology, biotechnology, biomanufacturing

## 1 Introduction

Biotechnology is a key enabler of a future bio-based and circular economy, supporting the sustainable production of chemicals, materials, fuels, and energy, while also enhancing human health through the production of pharmaceuticals and food ingredients (Yang et al., 2017; Lokko et al., 2018; Kim et al., 2023). Compared to petrochemical or thermochemical processes, bioproduction systems are generally more environmentally friendly, as they operate under milder conditions, generate less waste, and often use renewable or waste-derived feedstocks. Beyond Earth, biotechnology is also crucial for future space biomanufacturing (Berliner et al., 2022; Vengerova et al., 2024), providing essential goods and services for human space exploration while minimizing resource transportation from Earth.

Nevertheless, optimizing and controlling bioprocesses remain challenging due to the inherent complexity of cells, the *catalysts* in cell-based biomanufacturing. Cells exhibit *multiscale*, *multirate*, *nonlinear*, and *uncertain* dynamics (Ullah and Wolkenhauer, 2011; Glass et al., 2021; Luo et al., 2021; Hartmann et al., 2022), which are nontrivial to capture in mathematical models. Uncertainties arise from factors such as stochasticity in gene expression and reaction networks, as well as environmental disturbances, all of which can lead to suboptimal and inconsistent bioprocess performance if not effectively addressed. These complexities and uncertainties ultimately limit the competitiveness of biotechnologies and hinder their broader commercial and industrial adoption.

Two major systems disciplines support biotechnology. On one hand, Systems Biology (SB) provides mathematical and computational methods to understand biological phenomena across different omics levels and scales (e.g., genomics, transcriptomics, proteomics, fluxomics, and metabolomics) (Noble, 2010; Otero and Nielsen, 2010; Kildegaard et al., 2013; Bellomo et al., 2015; Tavassoly et al., 2018). This field is supported by scientists with backgrounds in different areas, including biology, bioinformatics, mathematics, and physics. On the other hand, Process Systems Engineering (PSE) focuses on mathematical modeling and computer-aided methods for the design, optimization, and control of *production* processes, typically at a macroscopic scale, considering process-level material and energy balances (Pistikopoulos et al., 2021; Vassiliadis et al., 2024; Daoutidis et al., 2024). PSE finds its roots mainly in the chemical and process engineering communities.

Although PSE provides powerful methods for modeling, optimization, and control of complex dynamic systems, its application to biotechnology is often restricted to the macroscopic bioreactor level. For instance, mathematical models frequently oversimplify metabolic pathways by lumping them into macroscopic and phenomenological structures (Muloiwa et al., 2020; Straathof, 2023). As such, this neglects the intracellular domain and its potential degrees of freedom for advanced applications, such as the dynamic fine-tuning of metabolic fluxes to manage intrinsic metabolic trade-offs (e.g., growth vs production *modes*). Conversely, while SB enhances our understanding of cellular processes and enables rational genetic and metabolic engineering interventions for designing cell factories (Von Kamp et al., 2017; Schneider et al., 2020; Foster et al., 2021; Bekiaris and Klamt, 2021), a critical gap remains between cell factory design and process-level and plant-wide optimization and control.

In addition, we are currently in the era of the Fourth Industrial Revolution (Industry 4.0), where the goal is to fully digitalize and automate industrial processes by leveraging advanced modeling, real-time learning, and adaptive model-based optimization and control (Pandey et al., 2024; Isoko et al., 2024). In this context, this opinion paper highlights the need for the next generation of bioengineers to adopt a *systems-of-systems* perspective to bridge existing gaps between SB and PSE, enabling biotechnology to align with Industry 4.0 and fully realize its potential. Achieving this requires integrating SB and PSE into a unified framework, potentially leading to the formalization of a new discipline and scientific community: Biotechnology Systems Engineering (BSE). This paradigm shift entails fostering interdisciplinary education, curriculum development, and dedicated publication and conference platforms to support its growth and consolidation. However, formalizing such a holistic framework presents challenges, including differences in scope and research cultures, ranging from an explanatory-driven and mechanistic-oriented focus of SB to an application-driven and performance-oriented focus of PSE.

## 2 Discussion

**FIGURE 1:**
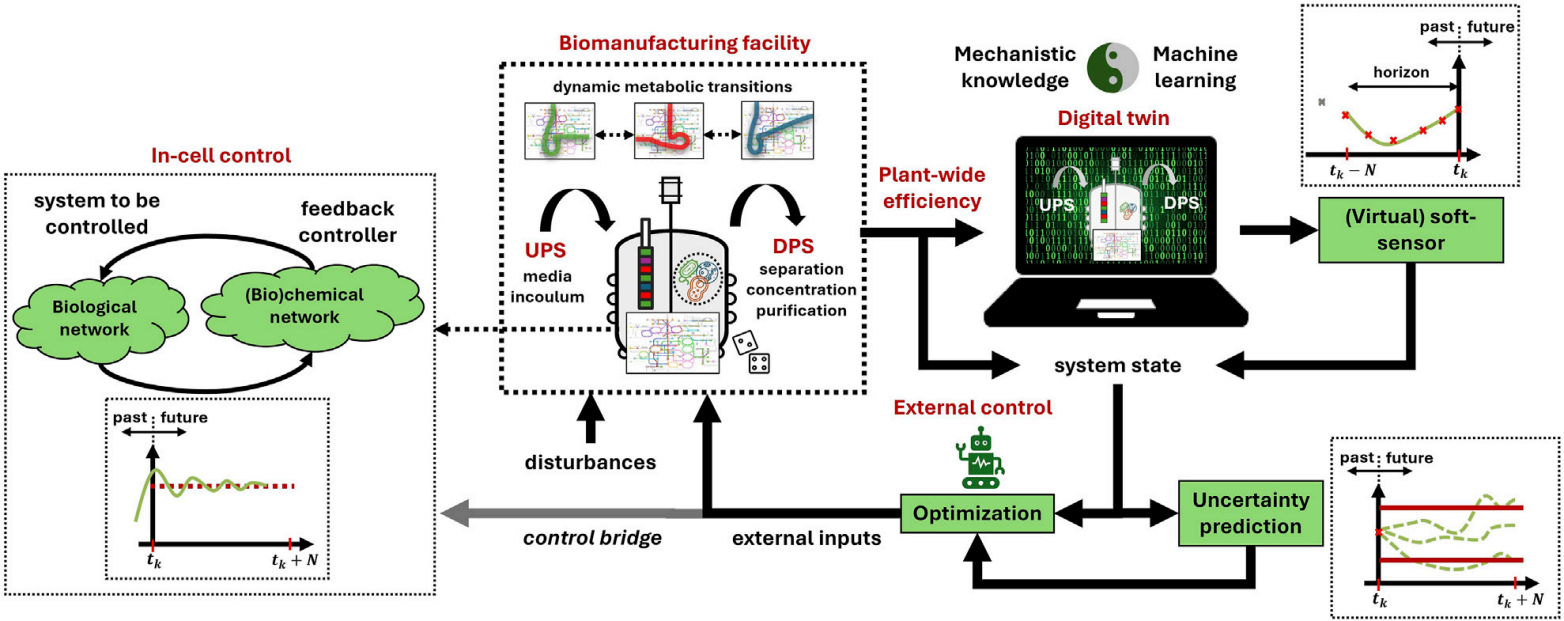
Overview of Biotechnology Systems Engineering (BSE) as a unifying framework integrating Systems Biology (SB) and Process Systems Engineering (PSE). The scheme focuses on multi-scale modeling and multi-level control in bioprocesses with plant-wide awareness and under uncertainty. The ultimate goal of BSE is to optimize plant-wide efficiency through adaptive learning, continuous model updating, and self-adaptive optimization and control. Digital twins, supported by mathematical modeling, integrate mechanistic approaches with machine learning to enhance model generalization and predictive capabilities. BSE models link bioreactor dynamics to the overall economic and sustainability aspects of the biomanufacturing facility. Multi-scale control synergistically integrates external controllers with in-cell controllers encoded by biochemical networks, aiming to maximize the efficiency of metabolism in the context of the plant-wide performance. Soft sensors and biosensors further support state estimation when only partial measurements are available. UPS: upstream processing, DPS: downstream processing.

This section first outlines the core capabilities of SB and PSE for biotechnological production. Then, an overview of the BSE framework is presented (cf. Figure 1), along with key potential actions to formalize it as a new discipline and community.

### 2.1 Systems Biology

The rise of high-throughput experimental platforms has moved biotechnology into the domain of *big data* (Shukla et al., 2022). Multi-omics data plays a crucial role in constructing and validating mathematical models in SB (Cotten and Reed, 2013; Kim et al., 2016; Kong et al., 2024). Genomics defines the cell’s metabolic potential, determining which enzymes can, in principle, be synthesized. Transcriptomics provides insights into regulatory mechanisms that influence enzyme expression. Proteomics quantifies enzyme abundance, directly linking metabolic fluxes to catalytic capacity. Fluxomics quantifies metabolic fluxes across metabolic pathways, giving information on the cell’s metabolic flux distribution. Finally, metabolomics determines intracellular metabolite concentrations, offering insight into the dynamics of metabolic intermediates.

Mathematical models of metabolism in SB often begin with genome-scale or reduced stoichiometric networks that link genes, proteins, and reactions (Cotten and Reed, 2013; Erdrich et al., 2015; Kim et al., 2016; Kong et al., 2024). The stoichiometric network serves as a foundation for building mass balances of metabolites, where metabolic fluxes function as reaction rates. A fundamental challenge is the parameterization of these metabolic fluxes, leading to two distinct modeling approaches: 1) constraint-based modeling and 2) kinetic modeling.

Constraint-based modeling treats metabolic fluxes as *decision variables* in a biologically inspired optimization problem, addressing system underdetermination (i.e., more unknowns fluxes than mass-balance equations) (Mahadevan et al., 2002; Gottstein et al., 2016; Volkova et al., 2020; Moulin et al., 2021). This optimization considers a biologically relevant objective function, such as maximizing growth, and is subject to biological and physiological constraints, with mass-balance constraints at its core. Often solved under pseudo-steady-state assumption of intracellular metabolism, constraint-based modeling provides a *snapshot* of the metabolic flux distribution for a given *temporal* metabolic state. This approach can be adapted to capture dynamic cellular behavior, e.g., of biomass and extracellular species, by discretizing a dynamic optimization problem over time or approximating local fluxes at discrete time points.

In contrast to constraint-based approaches, kinetic modeling explicitly describes fluxes as time-dependent functions governed by enzyme kinetics and metabolite concentrations (Van Rosmalen et al., 2021; Lu et al., 2021; Saa and Nielsen, 2017). This offers in principle more insight into the cell, capturing accumulation of both metabolic intermediates and extracellular species. However, such models are often highly nonlinear, which can be numerically difficult to handle (e.g., in model-based optimization and control tasks), and can make measuring states, as well as model parametrization, more challenging.

Computational methods based on constraint-based and kinetic modeling have enabled the identification of promising metabolic engineering interventions, such as gene deletions to enhance production pathway efficiency, addressing growth-production trade-offs, and designing stable microbial consortia (Von Kamp et al., 2017; Schneider et al., 2020; Foster et al., 2021; Bekiaris and Klamt, 2021). Additionally, mechanistic models in SB provide valuable insights into metabolic pathway utilization. For instance, they can predict metabolic flux distributions under specific conditions (Gerken-Starepravo et al., 2022; Pennington et al., 2024; Boecker et al., 2021; Boecker et al., 2022) and aid in identifying potential dynamic metabolic control strategies, where key fluxes are subjected to modulation (Jabarivelisdeh and Waldherr, 2018; Boecker et al., 2021; Espinel-Ríos et al., 2022a; Espinel-Ríos and Avalos, 2024b). Machine learning can be incorporated or assist in both constraint-based and kinetic modeling for more efficient model parameterization and enhanced predictability, as well as to facilitate and guide strain design (Li et al., 2022; Kundu et al., 2024; Choudhury et al., 2024).

### 2.2 Process Systems Engineering

PSE in biotechnology generally emphasizes macroscopic and environmental variables such as feed rates, oxygen availability, light, temperature, and pH, which can serve as degrees of freedom or targets in process optimization and control (Mears et al., 2017; Petsagkourakis et al., 2020; Treloar et al., 2020; Harcum et al., 2022; Chai et al., 2022; Espinel-Ríos et al., 2023a; Espinel-Ríos et al., 2024a; Espinel-Ríos et al., 2024b; Hoffman et al., 2025; Lee et al., 2025). These manipulatable variables often aim to maintain favorable growth and production conditions, or induce specific metabolic states. Various control strategies, including proportional-integral-derivative (PID) control, model predictive control (MPC), and reinforcement learning (RL), have been employed in those contexts.

In brief, PID control adjusts system inputs using proportional, integral, and derivative gains based on the tracking error (Åström and Murray, 2021). MPC, in contrast, employs a dynamic model of the system to solve a sequence of constrained open-loop optimizations based on the current system state (Rawlings et al., 2020). RL, a machine-learning-based control approach, enables an agent to iteratively learn an optimal control policy by interacting with the process and maximizing performance through reward-based feedback (Sutton and Barto, 2018).

It is worth mentioning that PSE increasingly incorporates *uncertainty-aware* and *adaptive* modeling, optimization, and control strategies toward enhancing robustness (Petsagkourakis et al., 2020; Morabito et al., 2021; Morabito et al., 2022; Espinel-Ríos et al., 2023b; Pennington et al., 2024; Espinel-Ríos et al., 2025). Additionally, soft or virtual sensors play a crucial role in PSE by enabling real-time state estimation using only a subset of available measurements (Randek and Mandenius, 2018; Elsheikh et al., 2021; Espinel-Ríos et al., 2022b; Espinel-Ríos et al., 2023c; Espinel-Ríos et al., 2024; Espinel-Ríos and Avalos, 2024a). More recently, machine-learning approaches have been incorporated into macroscopic and phenomenological models, as typically used in PSE, to create hybrid models (Tsopanoglou and Jiménez Del Val, 2021; Agharafeie et al., 2023; Mahanty, 2023). These *gray-box* models help to alleviate the negative effects of possible model oversimplifications, wrong model assumptions, and in general the lack of insight into intracellular mechanisms.

PSE also addresses the techno-economic feasibility and life-cycle assessment of biomanufacturing facilities, typically based on macroscopic mass and energy balances across unit operations (Fu et al., 2023; Wowra et al., 2023; Malinov et al., 2024). This broader perspective acknowledges that a bioprocess factory extends beyond the bioreactor and requires the integration of upstream and downstream processes, which ultimately determine its viability and sustainability. Upstream operations include media preparation and inoculum development, while downstream processes involve product separation, concentration, and purification.

### 2.3 Enter Biotechnology Systems Engineering

To recapitulate, PSE and SB have traditionally operated as separate paradigms in biotechnology, largely due to their origins in distinct scientific communities. PSE prioritizes the optimal operation of the production facility, with the bioreactor at the core, and extends to plant-wide techno-economic feasibility and life-cycle assessment. In contrast, SB typically focuses on local cellular objectives, such as maximizing the flux through the product-of-interest pathway facilitated by metabolic network engineering.

BSE aims to establish an integrated framework that merges SB and PSE to fully exploit biotechnology’s potential. This shift requires moving beyond purely intracellular, process-level, or plant-wide models toward multi-scale modeling schemes that effectively integrate these domains. To this end, BSE should drive the development of mathematical models incorporating, e.g., gene expression, resource allocation, regulatory mechanisms, and metabolic reactions while linking cellular behavior and phenotypes to varying external control inputs, process operating conditions, bioreactor designs, initial conditions, process disturbances, and system uncertainties. This holistic integration is essential for developing predictive and generalizable bioprocess digital twins, unlocking new degrees of freedom across scales for advanced bioprocess design, optimization, and control. In this regard, model reduction techniques (Ali Eshtewy and Scholz, 2020; Van Rosmalen et al., 2021) can assist in the development of digital twins, rendering smaller models while preserving the information and knowledge from the larger-order system.

Leveraging multi-scale modeling and digital twins, BSE should also drive the development of multi-level control strategies, integrating external process control (e.g., MPC or RL) with in-cell control mechanisms. Unlike traditional external control approaches in PSE, in-cell control relies on intracellular regulatory systems, such as (bio)chemical reaction networks, to encode control-like behavior (e.g., PID-like control) without user intervention after deployment. These controllers operate with *instantaneous* feedback and response, leveraging the intrinsic speed of chemical reactions, and have been shown to be robust against noisy biological networks (Chevalier et al., 2019; Filo et al., 2023; Alexis et al., 2024).

In this multi-level control framework, external control serves a supervisory role, dynamically adjusting setpoints for in-cell controllers or correcting deviations in their performance. This is particularly relevant when real-time monitoring and targeted actuation within intracellular networks may not be feasible. Therefore, such tasks can be delegated to in-cell controllers. Genetically encoded biosensors (e.g., fluorescence-based) can facilitate the implementation of such multi-level control strategies by providing real-time monitoring of cellular wellbeing, pathway utilization, or protein expression (Polizzi and Kontoravdi, 2015; Thorn, 2017; Zhang et al., 2022).

Since metabolic rates determine the catalytic efficiency of bioprocesses, real-time modulation of the metabolic flux distribution, within the context of plant-wide efficiency, should be a key focus for multi-level controllers. For example, external controllers can dynamically adjust the setpoints of in-cell controllers that are designed to achieve robust gene expression of target metabolic enzyme levels over desired periods. This approach would synergistically integrate control frameworks such as metabolic cybergenetics (Carrasco-López et al., 2020; Espinel-Ríos et al., 2024; Espinel-Ríos and Avalos, 2024a) with in-cell controllers.

To fully realize the potential of multi-level controllers in BSE, experimental validation is essential. Thus, the ease of implementation should be assessed throughout the research and development process, and these controllers must be tested for robustness against system uncertainties and disturbances. However, this should not discourage research into novel, more theoretically driven control methodologies, even if their experimental implementation is nontrivial. In such cases, identifying and understanding the gaps to experimental implementation can increase the likelihood of future realization. I believe the stage for experimental realization of multi-level controllers in BSE is favorable given the previous success in the areas of synthetic biology and cybergenetics. For instance, following separate strategies in control design, the experimental implementation of in-cell controllers has demonstrated their feasibility in modulating gene expression (Aoki et al., 2019; Frei et al., 2022), while external controllers have also been validated in both open-loop (Espinel-Ríos et al., 2024a) and closed-loop systems (Milias-Argeitis et al., 2016; Gutiérrez Mena et al., 2022) for similar purposes.

In the spirit of democratizing access to knowledge, BSE should prioritize open-source computational tools and bioreactor systems. This would empower institutions worldwide, particularly in developing regions, to advance research and innovation in BSE. These efforts promise to accelerate the development of bio-based technological platforms, fostering the widespread adoption of biotechnologies and ultimately contributing to global economic growth and sustainability. There are ongoing initiatives that embrace ideas related to knowledge democratization and modeling standardization in SB, from which BSE could benefit and build upon. For example, the Systems Biology Markup Language (SBML) is a standard format for exchanging biological models (Keating et al., 2020). Similarly, the CURE principles (Credibility, Understandability, Reproducibility, and Extensibility) advocate for better practices in biological modeling (Sauro et al., 2025).

To establish BSE as a field, undergraduate and graduate curricula should explicitly and purposely integrate SB and PSE in a holistic manner. Open-source computational tools should play a central role in education, enabling students to design, implement, and test a wide range of modeling, optimization, and control strategies in a cost-effective and safe environment. These tools would provide an affordable alternative to traditional wet-lab experimentation, which can be impractical or prohibitively expensive in some contexts. Yet, hands-on experimental work remains invaluable for bridging the gap between theory and practice. For example, open-source and accessible bioreactor systems, such as Chi.Bio platforms (Steel et al., 2020), could offer a powerful learning environment by allowing direct integration with user-coded software. Furthermore, BSE could benefit from existing open-source process simulators and techno-economic analysis software, such as BioSTEAM (Cortes-Peña et al., 2020), to facilitate innovation in sustainable process design when dealing with biotechnological production systems.

In addition, the educational approach in BSE should foster a generalist mindset. As an *engineering* discipline, BSE’s main driver should be the pursuit of *technological innovation* and *problem-solving*. Thus, a key focus should be on developing integrated frameworks that maximize the efficiency and productivity of bioproduction systems. While specialists are crucial for advancing their respective fields, BSE should aim to bridge disciplines by identifying gaps and integrating methodologies in ways that specialists may not immediately consider. In this context, I envision BSE playing a pivotal role in generating future leaders who will drive biotechnology to the next level of competitiveness, business innovation, and technological readiness.

BSE would benefit from specialized publication avenues and dedicated conferences, providing forums for collaboration and knowledge exchange. Additionally, BSE-focused student competitions, mirroring synthetic-biology-driven initiatives like iGEM (International Genetically Engineered Machine) (Zhan et al., 2023), could boost innovation and out-of-the-box thinking in bioproduction, while proactively incorporating BSE principles. These competitions could follow a two-stage structure, beginning with simulation-based approaches and progressing to hardware-based implementations, supported by accessible open-source bioreactors and funding for selected teams. Universities and the private sector could further support these initiatives by offering infrastructure, mentorship, and technical resources. Such competitions should have differentiated tracks, e.g., a scientific track focused on advancing methods and working frameworks, and an entrepreneurial track aimed at business development and fostering startup creation. This structure would provide an inclusive environment for people with different interests.

Finally, to some extent, other researchers and academics have also proposed systems-of-systems frameworks with motivations aligned to BSE (cf. e.g., (Koutinas et al., 2012; Kiss et al., 2015; Ko et al., 2020; Carrasco-López et al., 2020; Ohkubo et al., 2024)), indicating that this paradigm shift is already underway. Developing BSE methodologies is a key focus of my ongoing research. I strongly believe that equipping the next generation of bioengineers with a systems-of-systems framework as in BSE has the potential to revolutionize biotechnology.
